# Health-Related Quality of Life of Moroccan COVID-19 Survivors: A Case-Control Study

**DOI:** 10.3390/ijerph19148804

**Published:** 2022-07-20

**Authors:** Asmaa Azizi, Doha Achak, Elmadani Saad, Abderraouf Hilali, Chakib Nejjari, Mohamed Khalis, Ibtissam Youlyouz-Marfak, Abdelghafour Marfak

**Affiliations:** 1 Laboratory of Health Sciences and Technologies, Higher Institute of Health Sciences, Hassan First University of Settat, Settat 26000, Morocco; a.azizi@uhp.ac.ma (A.A.); d.achak@uhp.ac.ma (D.A.); saad.elmadani@uhp.ac.ma (E.S.); abderraouf.hilali@uhp.ac.ma (A.H.); ibtissam.marfak@uhp.ac.ma (I.Y.-M.); 2 International School of Public Health, Mohammed VI University of Health Sciences, Casablanca 82403, Morocco; cnejjari2000@yahoo.fr (C.N.); mkhalis@um6ss.ma (M.K.); 3 National School of Public Health, Ministry of Health, Rabat 10000, Morocco

**Keywords:** COVID-19, health related quality of life, EQ-5D-5L, SF-6D, anxiety, depression, pain

## Abstract

Background: Research on COVID-19 has mostly focused on transmission, mortality and morbidity associated with the virus. However, less attention has been given to its impact on health-related quality of life (HRQoL) of patients with COVID-19. Therefore, this study aimed to determine the demographic and clinical risk factors associated with COVID-19 and evaluate its impact on the HRQoL of COVID-19 survivors. Methods: A case-control study was carried out between September 2021 and March 2022 on 1105 participants. A total of 354 were COVID-19 survivors and 751 were the control group. The HRQoL was assessed using both EQ-5D-5L and SF-6D generic instruments. Results: The average age of all participants was 56.17 ± 15.46. Older age, urban area, tobacco use, presence of chronic diseases especially type 1 diabetes, kidney and cardiovascular diseases were significantly associated with COVID-19. The COVID-19 survivors had significantly lower HRQoL (EQ-VAS = 50.89) compared to the control group (EQ-VAS = 63.36) (*p*-value < 0.0001). Pain/ discomfort and anxiety/depression were the most negatively affected by COVID-19 (*p*-value < 0.0001). Conclusions: The findings from this study could help healthcare professionals and policy makers to better understand the HRQoL sequelae among the COVID-19 survivors and contribute to develop tailored interventions.

## 1. Introduction

On 12 March 2020, the Coronavirus 2019 (COVID-19) disease was recognized by the World Health Organization (WHO) as a world public health issue. As of 15 March 2022, the WHO had reported more than 456 million of confirmed cases and 6 million cumulative deaths worldwide [1]. In Morocco, nowadays the number of confirmed cases and deaths has reached 1,162,125 and 16,043, respectively [2].

COVID-19 has caused critical challenges for public health and clinical research worldwide. Since the emergence of the COVID-19 pandemic, many studies have been focused on clinical spectrum [3,4,5,6,7] and treatment of infected patients [8,9,10,11]. Overall, most studies have focused on transmission, morbidity and mortality related to the virus. Researchers have also studied the socio-economic effect of COVID-19 [12,13,14]. The impact of the pandemic on mental health, well-being, and lifestyle of adults, adolescents and children has been demonstrated, particularly during the period of home confinement used as measure to prevent the outbreak spared [15,16,17,18,19]. The sequelae of COVID-19 in surviving patients after hospitalization have been reported by other investigators. Huang and colleagues showed that COVID-19 survivors were mainly troubled with fatigue, muscle weakness, sleep difficulties and anxiety or depression [20]. Jacobs et al. showed that the most persistent symptoms at 35 days were fatigue, dyspnoea, and muscular pain [21]. Joint pain and chest pain have also been observed as sequelae of COVID-19 [22]. The COVID-19 sequalae could affect patient quality of life. Some theories have shown that socio-demographic and clinical factors can be the predictors of COVID-19 severity [23,24]. For this reason, it has become necessary to understand these factors and determine their impact on the COVID-19 survivor’s health related quality of life (HRQoL). The HRQoL is a multidimensional concept reflecting the patient’s physical, psychological, social, and emotional well-being. The assessment of HRQoL remains an essential element in evaluating post-treatment health status of COVID-19 patients. Indeed, the description of the COVID-19 patients’ health status and the associated factors are important information for healthcare providers to identify the vulnerable groups that need special attention during this epidemic and improve their HRQoL. Consequently, HRQoL has become a health priority and the ultimate goal of medicine, health decision-making and health economic evaluation.

However, to the best of our knowledge, in Morocco, until now, there is no study focused on the impact of COVID-19 on the HRQoL of infected patients. Accordingly, to meet this need, we conducted a case-control study to compare the HRQoL between the COVID-19 survivors and the control group of uninfected individuals. The EQ-5D-5L and SF-6D are the widely used instruments for assessing HRQoL [25,26]. In our study, we used for the first time the EQ-5D-5L and the SF-6D questionnaires among the COVID-19 survivors and the control group. Using the two questionnaires simultaneously allow to evaluate both the EQ-5D-5L dimensions and the SF-6D domains which contributes to better understand the impact of COVID-19 on the HRQoL of infected patients. Findings from this study could provide more evidence for health decision-makers and therefore contribute to set up adequate protocols for managing the post-COVID-19 and the possible future pandemics.

## 2. Materials and Methods

### 2.1. Study Design and Data Collection

**Figure 1:**
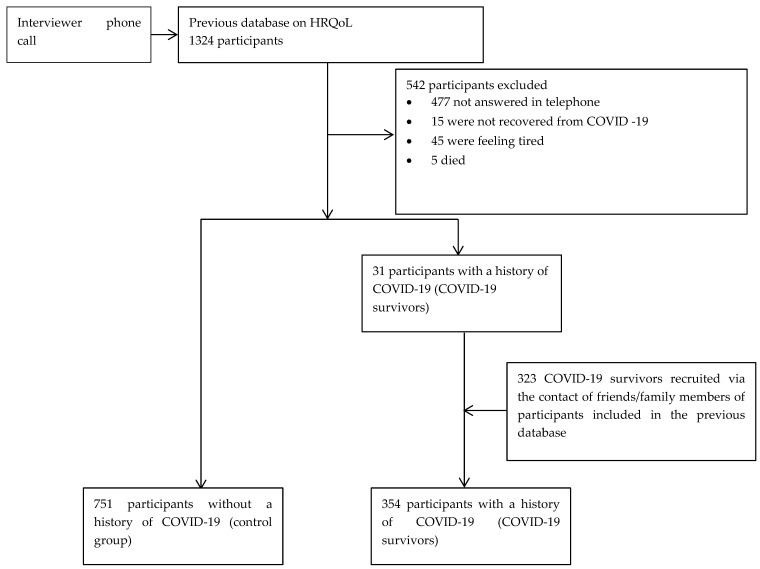
Flow chart of participants included in this study.

A case-control study was employed to assess the impact of COVID-19 on the HRQoL of the Moroccan infected patients between September 2021 and March 2022. This study included two groups: (1) COVID-19 survivors and (2) a control group. The inclusion criteria for COVID-19 survivors were the following: (i) patient aged 18 years and above, (ii) who had a confirmed diagnosis of COVID-19 by PCR test, (iii) who had been hospitalized for COVID-19, recovered and discharged from hospital for more than two days and not more than 90 days, (iv) those agreeing to participate in this study with verbal consent, (v) who with no previous follow-up of any psychiatric and mental disorders, and (vi) who able to understand and speak the Moroccan dialect. The exclusion criteria for COVID-19 survivors included, (i) patient under 18 years of age, (ii) patient who not yet recovered from COVID-19, (iii) patients with a previous follow-up of any psychiatric and mental disorders, and (iv) patient who did not give the verbal consent to participate in this study. Whereas, for the control group, the inclusion criteria consisted of (i) subject aged 18 years and above, (ii) who have never been diagnosed with the COVID-19, (iii) subjects with no previous follow-up of any psychiatric and mental disorders, (iv) who giving their informed consent to participate in this study, and (v) who able to understand and speak the Moroccan dialect. Figure 1 illustrates the data collection protocol of this case-control study. Using our previous HRQoL database of 1324 participants aged 18 years old and over, interviewers conducted a telephone conversation with each participant doing the following: (1) at the beginning of each conversation, the interviewers recalled the objectives of our previous study, (2) explained the purpose of this second data collection and (3) asked for oral consent. Next, (4) the interviewers asked whether the participant had contracted COVID-19 since the start of the pandemic. The participant was classified as a COVID-19 survivor if he/she responded that he/she had been diagnosed with COVID-19 and hospitalized. Otherwise, the participant was classified in the control group. The conversation then continued (5) with the collection of responses to the SF-6D and EQ-5D-5L questionnaires and the updating of socio-demographic data. (6) At the end of the conversation, the interviewer asked the participant to provide contact information for a family member/friend who was diagnosed and hospitalized with COVID-19 between March 2020 and December 2021. An interviewer subsequently conducted a telephonic interview with this individual basing on steps (2)–(6). A total of 1105 subjects participated in this study (354 cases and 751 controls).

### 2.2. Measurements

Socio-demographic and clinical characteristics (age, gender, marital status, number of children, education level, employment status, place of residence, and socio-economic level, tobacco use, presence of chronic disease and type of chronic diseases) were collected. Health related quality of life was assessed using the EQ-5D-5L and SF-6D generic instruments.

The EQ-5D-5L instrument consists of a descriptive system and a Visual Analog Scale (VAS) [25]. The descriptive system comprises five dimensions (5D) including (i) mobility, (ii) self-care, (iii) usual activities, (iv) pain/discomfort and (v) anxiety/depression. For each dimension there are five levels (5L) to represent the degree of the health state severity: no problems (level 1), slight problems (level 2), moderate problems (level 3), severe problems (level 4) and extreme problems (level 5). The participant’s response was converted to a five-digit number describing the health state, i.e., 13,524 is the health state equivalent to no problems in mobility, moderate problems in self-care, extreme problems in usual activities, slight problems in pain/discomfort, and severe problems in anxiety/depression. The VAS was used to assess subjects health status with a component score from 0 to 100, where 0 refers to a worse imaginable health state and 100 to a best imaginable heath state.

The SF-6D instrument was developed from Short Form 36 by Brazier et al. [24], then translated and validated in Arabic [27]. It was composed of a total of 31 items constituting six dimensions: (i) physical functioning, (ii) role limitation, (iii) social functioning, (iv) pain, (v) mental health, (vi) and vitality. Each of the six dimensions consisted of several levels ranging from four to six levels. The physical functioning and pain domain each consisted of six levels. The social function, the mental health and vitality domains consisted of five levels. The role limitation consists of four levels. The Cronbach’s alpha for the SF-6D Instrument used in this study was 0.96 which exhibiting a very good validity.

The average time required by each participant to complete the EQ-5D-5L and SF-6D generic measures was approximately 20 min.

### 2.3. Sampling

The aim of this case-control study was the assessment of health-related quality of life (HRQoL) in COVID-19 survivors, and given that anxiety is a dimension of HRQoL, we based the calculation of the number of subjects on the proportion of anxiety. Studies have assessed the level of anxiety during the period of COVID-19 confinement. In contrast, the level of anxiety in COVID-19 patients has not yet been estimated at the national level. In the present study, we assumed to detect at least a 10% difference in the level of anxiety between cases and controls. The minimum number of subjects was calculated from the following equation:

n=r+1rp*1−p*Zβ+Zα22ε2

where: r = control/case ratio, we consider two controls for one case. As the proportion of anxiety is not yet estimated, we assume p* = 0.5.

For a 95% confidence interval (α = 0.05) and statistical power of 80% (α = 0.2) and a minimum difference to be detected of = 10%, the minimum number of subjects to interview is n = 882 (294 cases and 588 controls).

### 2.4. Statistical Analysis

Categorical variables were reported as number and percentage, while continuous variables were summarized by means and standard deviation. For the association between socio-demographic/clinical characteristic and COVID-19, a binary logistic regression analysis was conducted. The comparison of HRQoL dimensions between COVID-19 survivors and the control group was tested by Mann–Whitney U test. To study the association between socio-demographic/clinical variables and EQ-VAS score, the Mann–Whitney U and the Kruskal–Wallis tests were performed for two and multiple comparisons, respectively. For the COVID-19 survivors’ group, a multiple linear regression was used to regress the HRQoL on socio-demographic and clinical variables. Correlation between dimensions of the EQ-5D-5L and SF-6D were assessed using Spearman’s correlation coefficient for both COVID-19 survivors and non-COVID-19. All statistical analysis was performed using the R software (version 4.0.3). Statistical tests were carried out with a significant level α = 0.05 and a 95% confidence interval.

## 3. Results

A total of 1105 subjects participated in this study. Among them, 751 were without history of COVID-19 (control group) and 354 were COVID-19 survivors (cases: 31 participants from the previous HRQoL database and 323 recruited via the contact given by friend/family members of participants interviewed from database). The percentage of COVID-19 from the database was 2.3% (31/1324) (Figure 1).

### 3.1. Association between COVID-19 and Socio-Demographic and Clinical Characteristics

The socio-demographic and clinical characteristics of cases and the controls are shown in Table 1. The mean age of all participants was 56.17 ± 15.46 with 51.6% female. Regarding marital status, 69% were married. Most participants (88.1% and 77.2% from COVID-19 survivors and the control group, respectively) had children. Four hundred ninety-six (44.9% from both groups) were illiterate. In relation to employment status, 33.8% was employed. About 62% of participants resided in urban areas. In relation to the socio-economic level, 55.6% were in a medium-income. One hundred and forty-one (12.8%) were smokers. Regarding the clinical characteristics, 51.7% from both groups suffered from chronic diseases. Diabetes mellitus, hypertension, kidney disease and cardiovascular disease were observed in 35%, 28%, 31% and 9% of participants, respectively. The unadjusted odds ratios showed that gender, education level, employment status, socio-economic level and hypertension were not significantly associated with COVID-19 (Table 1).

Table 2 shows the results of the multivariable binary logistic regression analysis. Seven variables (age, place of residence, tobacco use, presence of chronic diseases, type 1 diabetes, kidney, and cardiovascular diseases) were significantly associated with COVID-19. The adjusted odds of COVID-19 were two times higher among participants aged between 41–60 and 60 years old and above than younger (18–40 years). Participants living in rural area had lower odds of COVID-19 (0.07 [0.05–0.11], *p* < 0.0001) compared to urban participants. Regarding the clinical characteristics, the results showed that there was a significant association between the presence of chronic diseases and COVID-19 (odds = 4.28 [2.80–6.55], *p* < 0.0001). More specifically, the odds of COVID-19 were three times higher among patients with type 1 (3.60 [2.39–5.41], *p* < 0.0001), about two times higher among patients with kidney disease (1.67 [1.08–2.60], *p* = 0.021) and five times in patients with cardiovascular diseases (4.93 [2.68–9.07], *p* < 0.0001) than healthy participants (Table 2).

### 3.2. Health-Related Quality of Life of the COVID-19 Survivors versus Control Group

**Figure 2:**
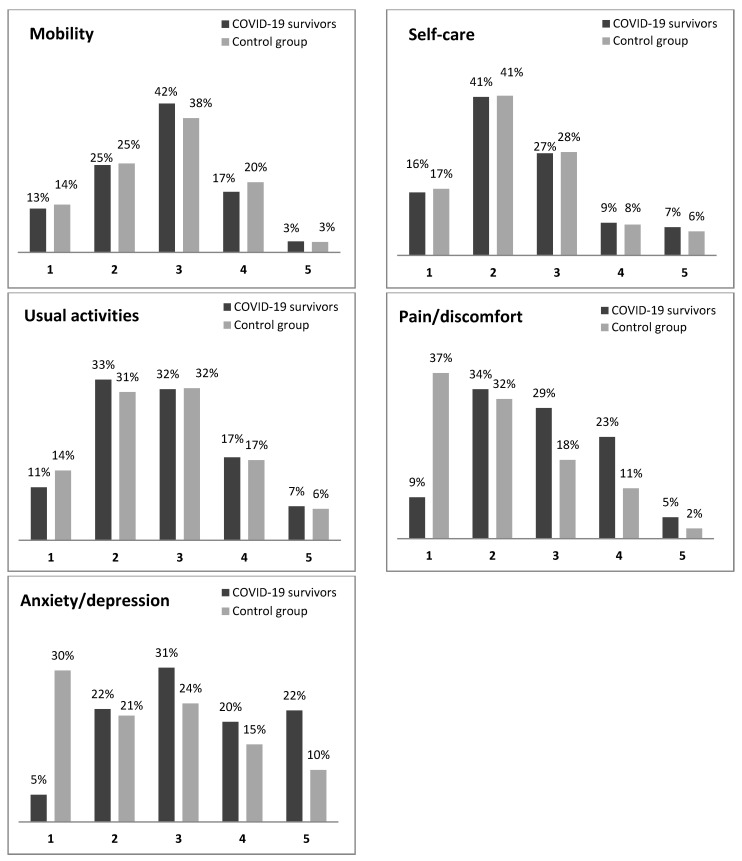
Comparison of each EQ-5D-5L dimension among COVID-19 survivors versus the control group.

The EQ-VAS scores showed that the COVID-19 survivors had significantly lower HRQoL (VAS = 50.89) compared to the control group (VAS = 63.36) (*p* < 0.0001). Figure 2 shows the EQ-5D-5L response distributions for the five health dimensions of COVID-19 survivors and the control group. For the mobility, self-care and usual activities dimensions, no significant differences were observed. Conversely, for pain/discomfort dimension, the Mann–Whitney U test demonstrated that COVID-19 survivors were observed to have more pain/discomfort than the control group (*p* < 0.0001). Furthermore, anxiety/depression dimension was affected by COVID-19 infection. In fact, the proportion of participants that reported having no anxiety/depression was six times less for COVID-19 survivors than the control group (*p* < 00001).

**Figure 3:**
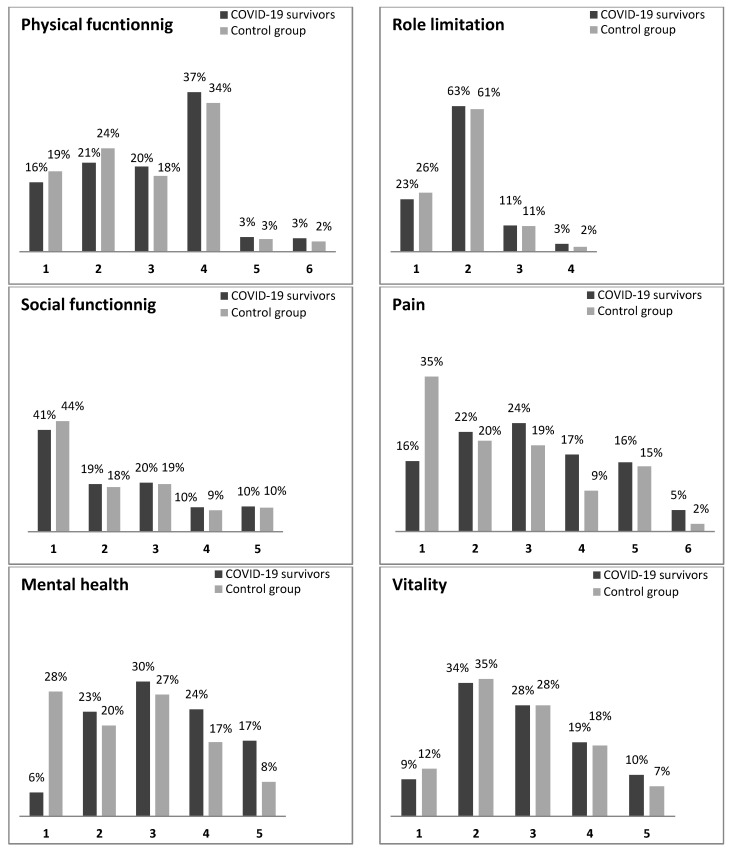
Comparison of each SF-6D dimension among COVID-19 survivors versus the control group.

The distribution of HRQoL problems reported by participants for each SF-6D dimensions is shown in Figure 3. We observed a negative effect of the COVID-19 on pain, and mental health (*p* < 0.0001). Indeed, the proportions of COVID-19 survivors reporting extreme problems for pain and mental health were respectively 5% and 17% compared to 2% and 8% for the control group. Whereas there was no significant impact of COVID-19 on physical functioning, role limitation, social functioning, and vitality.

### 3.3. Comparison of EQ-5D-5L and SF-6D Dimensions between COVID-19 Survivors and the Control Group Stratified on Socio-Demographic and Clinical Variables

Table 3 and Table 4 summarize the HRQoL of the COVID-19 survivors and the control group stratified by socio-demographic and clinical variables. From the EQ-5D-5L data, the Mann–Whitney U test showed that for both sexes and for all age categories, COVID-19 survivors had significantly higher level of pain/discomfort and anxiety/depression than the control group. These results were also confirmed by the SF-6D where COVID-19 survivors had poor mental health and higher level of pain than the control group. Concerning the marital status, single COVID-19 survivors were the most affected by COVID-19. Indeed, they reported having more problems for all dimensions of the EQ-5D-5L and SF-6D compared to single participants in the control group. We noted that all SF-6D and EQ-5D-5L dimensions were significantly affected among the COVID-19 survivors with the university educational level and, in comparison to the control group. Urban COVID-19 participants had significantly higher Mann–Whitney U mean rank on all HRQoL dimensions than those in the control group. However, only pain/discomfort, anxiety depression and mental health dimensions were affected by COVID-19 among participants living in rural areas. Furthermore, our results revealed that the COVID-19 survivors that were smokers and those with medium socio-economic classification reported having more problems in all EQ-5D-5L and SF-6D dimensions than those in the control group. Furthermore, our results showed that the COVID-19 survivors with chronic diseases had more pain/discomfort and anxiety/depression than those in the control group. These findings are also confirmed by the results of the SF-6D.

### 3.4. The COVID-19 Survivors’ HRQoL

The results of the univariate analysis for the association between EQ-VAS scores and both socio-demographic and clinical factors among COVID-19 survivors showed that gender, presence of children, educational level, employment status, socio-economic level, and place of residence were not statistically associated with EQ-VAS. Therefore, these variables were not examined in the multivariable analysis. Multiple linear regression showed that the age, tobacco use, and presence of chronic diseases specially type 1 diabetes, kidney, and cardiovascular diseases were the most important predictors of poor HRQoL of COVID-19 survivors (Table 5).

### 3.5. Correlation between the SF-6D Domains and the EQ-5D-5L Dimensions of the COVID-19 Survivors and Non-COVID-19

From Table 6, the average correlations between SF-6D and EQ-5D-5L among the COVID-19 survivors and non-COVID-19 were r = 0.68 [min = 0.51; max = 0.95] and r = 0.53 [min = 0.27; max = 0.93], respectively. For non-COVID-19 participants, we observed moderate correlations. Stronger correlations were noted among the COVID-19 survivors. In addition, strong correlations were observed between similar dimensions such as SF-6D-Pain and EQ-5D-5L-Pain/Discomfort (r = 0.93) and SF-6D-Mental Health and EQ-5D-5L-Anxiety/Depression (r = 0.95), which demonstrate a strong convergent between SD-6D and EQ-5D-5L.

## 4. Discussion

Our study focused on the impact of COVID-19 on the HRQoL of infected survivors. Among the demographic risk factors, we observed that older age was correlated with higher risk of COVID-19. Similar findings have been reported by other studies [28,29]. Our results showed that there was no significant association between gender and the risk of COVID-19. Literature reported heterogeneity in this association. For example, Pietrobon et al. found that males are most susceptible to the COVID-19 infection [23], whereas Thai et al. observed a slight difference between the gender proportions of COVID-19 [30]. Our study found that urban residence was also associated with a high risk of the COVID-19 infection. This is likely due to the high number of people living in urban areas and the high rate of daily contact. Our results revealed that tobacco use also appeared to be a risk factor for COVID-19. The association between tobacco and COVID-19 infection (OR > 1.05, *p* = 0.02) was also reported by Chadeau-Hyam et al. [31].

The multivariable binary logistic regression showed that there was a significant association between presence of chronic diseases and COVID-19. In fact, we observed that participants with comorbid chronic conditions had higher odds ratio (4.28) compared to healthy participants. This finding confirms that obtained by Ejaz et al. [32]. Additionally, we observed that the odds of COVID-19 were three times higher among patients with type 1 diabetes. Similar results were obtained by other researchers [33,34,35]. In a systemic review and a meta-analysis of prevalence and impact of cardiac injury on COVID-19, Fu and colleagues found that the proportions of cardiac injury were 22% amongst 6297 hospitalized patients with COVID-19 [36]. This ascertainment supports why cardiac patients had a higher odds ratio. Patients with kidney disease had higher risk for COVID-19 compared to healthy individuals (odds = 1.67). This was consistent with the findings of a recent retrospective study, which found a highest prevalence of kidney diseases among hospitalized COVID-19 patients (38%) [37].

Our research showed the impact of COVID-19 on the HRQoL of the COVID-19 survivors. Indeed, the comparison of HRQoL between COVID-19 survivors and the control group reported that COVID-19 survivors had lower scores of HRQoL (VAS = 49.82) compared to the control group (VAS = 63.36). The same observation was reported before by other studies, which revealed that COVID-19 has been associated with persistent pulmonary function deterioration, muscle weakness, higher level of fatigue, pain, depression, anxiety, vocational problems, and decreased quality of life to various degrees [20,21,22,32,38,39].

Multivariate analysis showed that older age is among the most important predictors of lower HRQoL in COVID-19 survivors. This finding was like recent studies in the literature, which revealed that older age was associated with lower Quality of Life (QoL) of patients [31,40]. Arab-Zozani et al. reported that the difference between the mean HRQoL scores among COVID-19 patients was significantly depending on comorbidity [38]. Additionally, Bajgain et al. found that the presence of any coexisting comorbidity increased the risk of severe COVID-19 complications [24]. We obtained similar results showing that diabetes mellitus, cardiovascular and kidney diseases were the important predictors for poor HRQoL (*p* < 0.05).

Health economic studies showed that comorbidities among COVID-19 patients significantly increased the cost of health care. Furthermore, advanced COVID-19 severity and older age were strongly associated with higher cost [41]. It has been reported that the hospital length of stay and intensive care unit admission increased the health system cost [42]. This emphasized the need for appropriate strategy to reduce the health care cost related to COVID-19. For example, Kohli and colleagues demonstrated the positive impact of the vaccination against SARS-CoV-2 on the health economics where the cost per quality-adjusted life-year (QALY) gained <$50,000 [43].

In a prospective cohort study of 183 COVID-19 patients, Jacobs and colleagues found that muscular pain was the persistent symptom at 35 days after hospitalization for COVID-19 infection [21]. Another study assessed the impact of COVID-19 on survivors and their family members showed that the ‘pain and discomfort’ was the most affected EQ-5D dimension among the COVID-19 survivors [44]. In the same way, Carfì et al. demonstrated that joint pain and chest pain were reported as sequelae of COVID-19 patients [22]. These outcomes were congruent with our results where the COVID-19 survivors severe pain/discomfort compared to the control group.

COVID-19 is highly infectious, therefore, to limit its spread, infected patients are isolated, which reduces their social interaction. This can negatively affect patient psychological well-being [45,46]. In our research, there was a strong significant difference in anxiety/depression dimension between COVID-19 survivors and the control group (*p* < 0.0001). We obtained identical result from the SF-6D data, which revealed that mental health was the most affected health dimension by COVID-19. Similar results were also reported by the literature [20].

On the other hand, we compared the data from the 31 patients that we recruited from the previous database on HRQoL before having COVID-19 to the dataset of the same patients after they recovered from COVID-19 using the EQ-5D-5L and SF-6D instruments. The results of the EQ-5D-5L instrument showed that participants post COVID-19 had lower mean EQ-VAS score (51.86; *p* < 0.0001) compared to pre COVID-19 (VAS = 60.48). The comparison of each EQ-5D-5L dimension separately showed that the patients had more pain/discomfort and anxiety/depression levels after recovering than before having the COVID-19 (*p* = 0.002). These findings were also confirmed by the results of the SF-6D, which demonstrated that pain and mental health dimensions were found to be negatively impacted by COVID-19 (*p* = 0.003). This suggests that the COVID-19 survivors have impaired physical and mental function that led to decreased HRQoL.

The correlation between the SF-6D domains and the EQ-5D-5L dimensions of the COVID-19 survivors and non-COVID-19 group highlights stronger correlations among the COVID-19 survivors between similar dimensions such as SF-6D-Pain and EQ-5D-5L-Pain/Discomfort (r = 0.93) and SF-6D-Mental Health and EQ-5D-5L-Anxiety/Depression (r = 0.95), which demonstrate a strong convergence between SD-6D and EQ-5D-5L.

Given the results of this study, we recommend that healthcare providers and decision-makers develop preventative strategies to reduce the COVID-19 impact on the HRQoL for COVID-19 survivors, especially older persons, and patients with chronic diseases such as type 1 diabetes, kidney, and cardiovascular diseases. Additionally, to develop rehabilitation programs for the post-COVID-19 patients to help them to restore a good HRQoL.

Some limitations in this study should be pointed out. The lack of information about the patient experiences during their stay in the hospital, a qualitative study could be conducted to have more information on the impact of COVID-19 on the quality of life of the COVID-19 survivors. We evaluated the HRQoL only once, three months after hospital discharge. Repeated measurements would help to understand the evolution of the impact of COVID-19 on the HRQoL.

Despite the above potential limitation, this study benefits from several strengths. This is the first case-control study in Morocco, which focused on the demographic and clinical risk factors associated with COVID-19, evaluated the impact of COVID-19 on the HRQoL and examined the predictors of lower HRQoL among COVID-19 survivors. Likewise, this study used a control group, which allows more understanding of the impact of COVID-19 on the HRQoL of COVID-19 survivors. Furthermore, we had 31 paired observations that permitted us to compare HRQoL before and after recovering from COVID-19 for the same participants. Furthermore, we used standardized valid instruments, i.e., EQ-5D-5L and SF-6D. Moreover, our previous HRQoL database provided a unique opportunity to collect many COVID-19 survivors from both urban and rural area of several regions in Morocco.

## 5. Conclusions

The present study showed that the main demographic and clinical factors that significantly increase the risk of COVID-19 were older age, urban area, tobacco use, presence of chronic diseases especially type 1 diabetes, kidney disease and cardiovascular disease. In addition, our findings suggest that COVID-19 survivors have impaired physical and psychological health dimensions that led to lower HRQoL compared to the control group.

This study provided information on the impact of COVID-19 on the HRQoL for Moroccan COVID-19 survivors. These findings may help healthcare professionals and decision-makers to better understand the consequences of COVID-19 on the HRQoL and therefore gear towards post-COVID-19 care and provide opportunities to apply tailored interventions for COVID-19 survivors especially vulnerable patients who present other risk factors that can better manage the post-COVID-19 impact and restore a good QoL.

## Figures and Tables

**Table 1:** Socio-Demographic and Clinical Characteristics of the COVID-19 Survivors and Control Group (n = 1105).

Variables	COVID-19 Survivors (n = 354)	Control Group (n = 751)	Unadjusted OR [95% CI]	*p*-Value
	n (%)	n (%)		
**Age**				
18–40 41–60 +60	25 (7.1) 138 (39.0) 191 (53.9)	134 (17.8) 311 (41.4) 306 (40.8)	1 2.38 [1.48–3.81] 3.35 [2.10–5.32]	**<0.0001** **<0.0001**
**Gender**				
Female Male	175 (49.4) 179 (50.6)	395 (52.6) 356 (47.4)	1 1.13 [0.88–1.46]	0.327
**Marital status**				
Single Married Widowed	33 (9.3) 248 (70.1) 73 (20.6)	137 (18.2) 515 (68.6) 99 (13.2)	1 2 [1.33–3.01] 3.06 [1.88–4.97]	**0.001** **<0.0001**
**Presence of children**				
No Yes	42 (11.9) 312 (88.1)	171 (22.8) 580 (77.2)	1 2.19 [1.52–3.15]	**<0.0001**
**Educational level**				
Illiterate Primaryschool Secondary school University	153 (43.2) 50 (14.1) 82 (23.2) 69 (19.5)	343 (45.7) 103 (13.7) 163 (21.7) 142 (18.9)	1 1.09 [0.74–1.60] 1.13 [0.81–1.56] 1.09 [0.77–1.54]	0.669 0.471 0.627
**Employment status**				
Employed Unemployed/retired	118 (33.3) 236 (66.7)	256 (34.1) 495 (65.9)	1 1.03 [0.79–1.35]	0.805
**Place of residence**				
Urban Rural	309 (87.3) 45 (12.7)	382 (50.9) 369 (49.1)	1 0.15 [0.11–0.21]	**<0.0001**
**Socio-economic level**				
Low Medium High	132 (37.3) 189 (53.4) 33 (9.3)	255 (33.9) 425 (56.6) 71(9.5)	1 0.86 [0.65–1.13] 0.90 [0.56–1.43]	0.272 0.649
**Tobacco use**				
No Yes	292 (82.5) 62 (17.5)	672 (89.5) 79 (10.5)	1 1.81 [1.26–2.59]	**0.001**
**Presence of chronic diseases**				
No Yes	77 (21.8) 277 (78.2)	457 (60.9) 294 (39.1)	1 5.59 [4.18–7.49]	**<0.0001**
**Types of chronic diseases**				
**Type 1 diabetes**				
No Yes	204 (57.6) 150 (42.4)	600 (79.9) 151 (20.1)	1 2.92 [2.22–3.85]	**<0.0001**
**Type 2 diabetes**				
No Yes	303 (85.6) 51 (14.4)	716 (95.3) 35 (4.7)	1 3.44 [2.19–5.40]	**<0.0001**
**Hypertension**				
No Yes	242 (68.4) 112 (31.6)	550 (73.2) 201 (26.8)	1 1.27 [0.96–1.67]	0.094
**Kidney diseases**				
No Yes	199 (56.2) 155 (43.8)	564 (75.1) 187 (24.9)	1 2.35 [1.80–3.07]	**<0.0001**
**Cardiovascular diseases**				
No Yes	292 (82.5) 62 (17.5)	713 (94.9) 38 (5.1)	1 3.98 [2.60–6.10]	**<0.0001**

OR: odds ratio, 95% CI: 95% confidence interval. Significant *p* values (*p* < 0.05) are bold.

**Table 2:** Multivariable Binary Logistic Regression of Socio-Demographic and Clinical Characteristics of the COVID-19 Survivors versus the Control Group.

Variables	COVID-19 Survivors (n = 354)	Control Group (n = 751)	Adjusted OR [95% CI]	*p*-Value
	n (%)	n (%)		
**Age**				
18–40	25 (7.1)	134 (17.8)	1	
41–60	138 (39.0)	311 (41.4)	1.95 [1.04–3.67]	**0.038**
+60	191 (53.9)	306 (40.8)	1.99 [1.01–3.92]	**0.047**
**Marital status**				
Single	33 (9.3)	137 (18.2)	1	
Married	248 (70.1)	515 (68.6)	1.27 [0.45–3.59]	0.656
Widowed	73 (20.6)	99 (13.2)	0.92 [0.30–2.81]	0.890
**Presence of children**				
No	42 (11.9)	171 (22.8)	1	
Yes	312 (88.1)	580 (77.2)	1.56 [0.61–3.95]	0.351
**Place of residence**				
Urban	309 (87.3)	382 (50.9)	1	
Rural	45 (12.7)	369 (49.1)	0.07 [0.05–0.11]	**<0.0001**
**Tobacco use**				
No	292 (82.5)	672 (89.5)	1	
Yes	62 (17.5)	79 (10.5)	2.28 [1.38–3.75]	**0.001**
**Presence of chronic diseases**				
No	77 (21.8)	475 (60.9)	1	
Yes	277 (78.2)	294 (39.1)	4.28 [2.80–6.55]	**<0.0001**
**Types of chronic diseases**				
**Type 1 diabetes**				
No	204 (57.6)	600 (79.9)	1	
Yes	150 (42.4)	151 (20.1)	3.60 [2.39–5.41]	**<0.0001**
**Type 2 diabetes**				
No	303 (85.6)	716 (95.3)	1	
Yes	51 (14.4)	35 (4.7)	1.27 [0.65–2.49]	0.479
**Kidney diseases**				
No	199 (56.2)	564 (75.1)	1	
Yes	155 (43.8)	187 (24.9)	1.67 [1.08–2.60]	**0.021**
**Cardiovascular diseases**				
No	292 (82.5)	713 (94.9)	1	
Yes	62 (17.5)	38 (5.1)	4.93 [2.68–9.07]	**<0.0001**

OR: odds ratio, 95% CI: 95% confidence interval. Significant *p* values (*p* < 0.05) are bold.

**Table 3:** Comparison of EQ-5D-5L dimensions between COVID-19 survivors and the control group stratified on socio-demographics and clinical variables.

Variables	Mobility			Self Care			Usual Activities			Pain/Discomfort			Anxiety/Depression		
	C+	C−	*p*-Value *	C+	C−	*p*-Value *	C+	C−	*p*-Value *	C+	C−	*p*-Value *	C+	C−	*p*-Value *
	Mean Rank	Mean Rank		Mean Rank	Mean Rank		Mean Rank	Mean Rank		Mean Rank	Mean Rank		Mean Rank	Mean Rank	
**Age**															
18–40 41–60 +60	83.98 216.05 242.87	79.26 228.97 252.83	0.623 0.310 0.425	90.20 215.22 247.94	78.10 229.34 249.66	0.189 0.261 0.892	103.62 214.94 240.82	75.59 229.46 254.11	**0.003** 0.254 0.296	125.24 266.97 292.59	71.56 206.37 221.79	**<0.0001** **<0.0001** **<0.0001**	129.76 269.68 273.32	70.72 205.18 233.82	**<0.0001** **<0.0001** **0.002**
**Gender**															
Female Male	282.41 271.93	286.87 266.02	0.755 0.661	280.21 281.54	287.84 261.19	0.594 0.127	285.29 279.45	285.59 262.24	0.983 0.206	345.87 343.60	258.75 229.99	**<0.0001** **<0.0001**	334.68 340.34	263.71 231.63	**<0.0001** **<0.0001**
**Marital status**															
Single Married Widowed	111.17 351.14 90.94	79.32 396.86 83.23	**0.001** **0.005** 0.289	105.88 361.33 93.55	80.59 391.96 81.30	**0.004** 0.059 0.098	114.91 349.71 94.91	78.42 397.55 80.30	**<0.0001** **0.003** **0.049**	140.55 443.47 106.06	72.24 352.40 72.08	**<0.0001** **<0.0001** **<0.0001**	138.80 436.68 96.99	72.66 355.67 78.76	**<0.0001** **<0.0001** **0.014**
**Presence of children**															
No Yes	131.00 427.17	101.11 456.90	**0.003** 0.084	129.79 436.61	101.40 451.82	**0.004** 0.378	138.51 430.02	99.26 455.37	**<0.0001** 0.145	168.23 526.55	91.96 403.44	**<0.0001** **<0.0001**	162.45 511.30	93.38 411.64	**<0.0001** **<0.0001**
**Educational level**															
Illiterate	237.19	253.54	0.215	246.84	249.24	0.858	240.43	252.10	0.383	288.61	230.61	**<0.0001**	267.02	240.24	**0.049**
Primary school	69.25	80.76	0.104	79.86	75.61	0.552	67.57	81.58	**0.049**	90.82	70.29	**0.004**	87.00	72.15	**0.042**
Secondary school	129.24	119.86	0.304	124.30	122.34	0.827	122.34	119.63	0.273	168.07	100.33	**<0.0001**	165.05	101.84	**<0.0001**
University	125.69	96.43	**0.001**	115.13	101.56	0.098	131.86	93.44	**<0.0001**	150.40	84.43	**<0.0001**	155.99	81.71	**<0.0001**
**Employment status**															
Employed Unemployed/retired	219.27 337.88	172.86 379.41	**<0.0001** **0.008**	206.96 355.18	178.53 371.16	**0.010** 0.319	220.86 343.92	172.12 376.53	**<0.0001** **0.042**	271.25 418.50	148.90 340.97	**<0.0001** **<0.0001**	274.79 400.08	147.27 349.75	**<0.0001** **0.002**
**Place of residence**															
Urban Rural	369.95 182.92	326.62 210.50	**0.003** 0.118	363.39 206.53	331.93 207.62	**0.028** 0.952	382.36 201.87	316.59 208.19	**<0.0001** 0.726	450.14 243.10	261.76 203.16	**<0.0001** **0.028**	433.72 244.44	275.04 202.99	**<0.0001** **0.024**
**Socio-economic level**															
Low Medium High	174.36 330.26 47.39	204.17 297.38 54.87	**0.008** **0.027** 0.210	180.52 332.17 47.71	200.98 296.53 54.73	0.073 **0.016** 0.244	179.81 334.03 49.24	201.35 295.70 54.73	0.062 **0.010** 0.428	217.29 411.95 58.32	181.95 261.05 49.80	**0.002** **<0.0001** 0.162	201.25 407.69 64.76	190.25 262.95 46.80	0.345 **<0.0001** **0.004**
**Tobacco use**															
No Yes	471.65 78.93	487.21 64.78	0.404 **0.032**	475.90 84.32	485.37 60.54	0.610 **<0.0001**	472.62 89.23	486.79 56.70	0.452 **<0.0001**	588.30 99.45	436.53 48.67	**<0.0001** **<0.0001**	560.45 103.48	448.63 45.51	**<0.0001** **<0.0001**
**Presence of chronic diseases**															
**Type 1 diabetes**															
Yes	171.70	130.44	**<0.0001**	168.21	133.91	**<0.0001**	165.58	136.51	**0.003**	192.14	110.13	**<0.0001**	194.78	107.51	**<0.0001**
**Type 2 diabetes**															
Yes	46.56	39.04	0.124	51.44	31.93	**<0.0001**	45.87	40.04	0.261	51.15	32.36	**<0.0001**	51.36	32.04	**<0.0001**
**Hypertension**															
Yes	161.32	154.59	0.497	165.26	152.40	0.212	162.08	154.17	0.434	196.52	134.98	**<0.0001**	187.79	139.85	**<0.0001**
**Kidney diseases**															
Yes	168.17	174.26	0.547	183.53	161.53	**0.033**	179.00	165.28	0.183	212.52	137.50	**<0.0001**	205.99	142.91	**<0.0001**
**Cardiovascular diseases**															
Yes	47.73	55.03	0.190	51.31	49.18	0.709	58.28	45.73	**0.027**	49.85	51.57	0.764	59.13	45.21	**0.012**

* Mann–Whitney U Test; C+: COVID-19 survivors; C−: Control group. Significant *p* values (*p* < 0.05) are bold.

**Table 4:** Comparison of SF-6D dimensions between COVID-19 survivors and the control group stratified by socio-demographical and clinical variables.

Variables	Physical Functioning			Role Limitation			Social Functioning			Pain			Mental Health			Vitality		
	C+	C−	*p*-Value *	C+	C−	*p*-Value *	C+	C−	*p*-Value *	C+	C−	*p*-Value *	C+	C−	*p*-Value *	C+	C−	*p*-Value *
	Mean Rank	Mean Rank		Mean Rank	Mean Rank		Mean Rank	Mean Rank		Mean Rank	Mean Rank		Mean Rank	Mean Rank		Mean Rank	Mean Rank	
**Age**																		
18–40 41–60 +60	99.12 235.62 239.23	76.43 235.62 255.10	**0.017** 0.224 0.215	105.76 218.13 242.04	75.19 228.05 253.35	**0.001** 0.371 0.295	65.80 195.52 278.32	82.65 238.08 230.70	0.051 **<0.0001** **<0.0001**	117.80 251.15 265.92	72.95 213.40 238.44	**<0.0001** **0.003** **0.034**	131.46 266.29 264.93	70.40 206.68 239.06	**<0.0001** **<0.0001** **0.044**	**108.74** **230.66** **235.54**	**74.64** **222.49** **257.40**	**<0.0001** 0.522 0.086
**Gender**																		
Female Male	289.21 286.66	283.85 258.62	0.712 **0.039**	282.42 285.01	286.86 259.45	0.734 **0.034**	288.03 259.45	284.38 262.80	0.797 0.249	313.03 322.68	273.31 240.51	**0.007** **<0.0001**	329.12 336.66	266.17 233.48	**<0.0001** **<0.0001**	289.74 287.84	283.62 258.03	0.673 **0.028**
**Marital status**																		
Single Married Widowed	125.74 368.86 88.66	75.81 388.33 84.91	**<0.0001** 0.235 0.613	114.48 383.85 73.58	78.52 381.11 96.03	**<0.0001** 0.849 **0.001**	89.14 373.69 92.51	84.62 386.00 82.07	0.581 0.447 0.164	134.64 406.05 98.52	73.66 370.42 77.64	**<0.0001** **0.032** **0.006**	137.79 430.17 96.76	72.91 358.80 78.93	**<0.0001** **<0.0001** **0.017**	117.05 368.98 88.83	77.90 388.27 84.78	**<0.0001** 0.238 0.584
**Presence of children**																		
No Yes	150.90 437.32	96.22 451.44	**<0.0001** 0.418	132.39 439.68	100.76 450.17	**0.001** 0.490	112.20 449.62	105.72 444.82	0.494 0.782	160.33 483.46	93.90 426.62	**<0.0001** **0.001**	163.17 502.90	93.20 416.16	**<0.0001** **<0.0001**	141.62 436.88	98.50 451.67	**<0.0001** 0.395
**Educational level**																		
Illiterate	232.62	255.58	0.086	230.78	256.40	**0.024**	239.63	252.46	0.344	260.83	243.00	0.192	264.60	241.32	0.087	232.43	255.67	0.083
Primary school	67.69	81.52	0.057	78.96	76.05	0.661	75.84	77.56	0.816	77.55	76.73	0.912	90.68	70.36	**0.005**	75.09	77.93	0.692
Secondary school	136.89	116.01	**0.023**	132.85	118.05	0.073	128.23	120.37	0.384	161.26	103.75	**<0.0001**	162.31	103.22	**<0.0001**	135.00	116.96	**0.049**
University	141.90	88.56	**<0.0001**	126.60	95.99	**<0.0001**	123.96	97.27	**<0.0001**	143.98	87.55	**<0.0001**	151.99	83.65	**<0.0001**	137.42	90.73	**<0.0001**
**Employment status**																		
Employed	243.67	161.61	**<0.0001**	225.57	169.95	**<0.0001**	234.53	165.82	**<0.0001**	259.58	154.28	**<0.0001**	269.88	149.53	**<0.0001**	228.17	168.75	**<0.0001**
Unemployed/retired	330.87	382.75	**0.001**	341.28	377.78	**0.007**	325.36	385.38	**<0.0001**	376.56	360.97	0.340	396.50	351.46	**0.005**	351.46	374.96	0.083
**Place of residence**																		
Urban Rural	376.94 199.78	320.98 208.44	**<0.0001** 0.632	372.03 207.38	324.95 207.51	**0.001** 0.992	380.88 176.99	317.79 211.22	**<0.0001** 0.062	423.95 215.16	282.94 206.57	**<0.0001** 0.642	425.86 249.37	281.40 202.39	**<0.0001** **0.011**	383.46 195.56	315.70 208.96	**<0.0001** 0.457
**Socio-economic level**																		
Low Medium High	177.45 349.78 47.32	202.57 288.70 54.91	**0.027** **<0.0001** 0.194	186.76 334.09 45.67	197.75 295.68 55.68	0.233 **0.005** 0.082	167.00 356.63 38.95	207.97 285.65 58.80	**<0.0001** **<0.0001** **0.001**	197.26 383.72 51.71	192.31 273.60 52.87	0.673 **<0.0001** 0.853	198.86 398.49 67.20	191.48 267.04 45.67	0.526 **<0.0001** **<0.0001**	176.45 343.46 54.48	203.08 291.51 51.58	**0.021** **0.001** 0.624
**Tobacco use**																		
No Yes	480.17 90.83	483.51 55.44	0.859 **<0.0001**	475.72 88.02	485.45 57.64	0.559 **<0.0001**	476.12 88.64	485.27 57.16	0.625 **<0.0001**	536.94 97.34	458.85 50.33	**<0.0001** **<0.0001**	553.25 103.52	451.76 45.47	**<0.0001** **<0.0001**	481.29 87.25	483.02 58.25	0.927 **<0.0001**
**Presence of chronic diseases**																		
**Type 1 diabetes**																		
Yes	179.68	122.51	**<0.0001**	154.43	147.60	0.356	161.45	140.62	**0.030**	190.46	111.80	**<0.0001**	195.66	106.63	**<0.0001**	166.92	135.19	**0.001**
**Type 2 diabetes**																		
Yes	46.05	39.79	0.177	49.49	34.77	**<0.0001**	49.00	35.49	**0.010**	48.04	36.89	**0.033**	51.15	32.36	**<0.0001**	46.62	38.96	0.130
**Hypertension**																		
Yes	168.33	150.69	**0.049**	168.72	150.47	**0.015**	155.34	157.93	0.804	177.45	145.60	**0.002**	185.13	141.32	**<0.0001**	167.83	150.97	0.098
**Kidney diseases**																		
Yes	174.94	168.65	0.380	180.13	164.35	**0.038**	138.76	198.64	**<0.0001**	194.93	152.08	**<0.0001**	213.00	137.10	**<0.0001**	190.19	156.01	**0.001**
**Cardiovascular diseases**																		
Yes	58.61	45.53	**0.024**	54.67	43.70	**0.007**	59.88	35.20	**<0.0001**	60.18	44.56	**0.007**	61.00	44.06	**0.002**	58.32	45.71	**0.026**

* Mann–Whitney U Test; C+: COVID-19 survivors; C−: Control group. Significant *p* values (*p* < 0.05) are bold.

**Table 5:** Multiple Linear Regression of the Association between EQ-VAS Score and Both Socio-Demographic and Clinical Factors among COVID-19 Survivors (n = 354).

Variables	EQ-VAS		
	β [95% CI]	*t*	*p*-Value
Age	−0.082 [−3.84; −0.66]	−2.779	**0.006**
Marital status	−0.016 [−2.20; 1.19]	−0.587	0.558
Tobacco use	−0.051 [−4.47; −0.15]	−2.106	**0.036**
Presence of chronic diseases	−0.457 [−21.71; −16.37]	−14.012	**<0.0001**
Type 1 diabetes	−0.253 [−10.97; −6.60]	−7.922	**<0.0001**
Type 2 diabetes	−0.025 [−4.50; 2.02]	−0.748	0.455
Hypertension	−0.015 [−3.13; 1.99]	−0.438	0.662
Kidney diseases	−0.398 [−16.51; −11.06]	−9.960	**<0.0001**
Cardiovascular diseases	−0.253 [−13.93; −8.98]	−9.114	**<0.0001**

R2 = 0.81; β standardized regression coefficient; 95% CI confidence interval 95%. Significant *p* values (*p* < 0.05) are bold.

**Table 6:** Spearman’s correlation coefficient between the SF-6D domains and the EQ-5D-5L dimensions of the COVID-19 survivors and non-COVID-19.

SF-6D	EQ-5D-5L				
	Mobility	Self-Care	Usual Activities	Pain/Discomfort	Anxiety/Depression
**COVID-19 survivors (n = 354)**					
Physical function	0.85 ***	0.71 **	0.70 **	0.71 **	0.75 ***
Role limitation	0.57 *	0.67 **	0.52 *	0.57 *	0.59 *
Social function	0.59 *	0.65 **	0.59 **	0.61 *	0.51 *
Pain	0.69 **	0.70 **	0.67 **	0.93 ***	0.72 **
Mental health	0.61 *	0.71 **	0.62 *	0.75 ***	0.95 ***
Vitality	0.64 **	0.68 **	0.69 **	0.66 **	0.75 ***
**Non-COVID-19 (n = 751)**					
Physical function	0.57 **	0.40 *	0.49 *	0.54 **	0.64 **
Role limitation	0.37 *	0.27 *	0.43 *	0.34 *	0.39 *
Social function	0.42 *	0.39 *	0.45 *	0.49 *	0.56 **
Pain	0.51 *	0.50 *	0.58 **	0.91 ***	0.65 **
Mental health	0.54 *	0.42 *	0.57 **	0.62 **	0.93 ***
Vitality	0.52 *	0.43 *	0.65 **	0.61 **	0.59 **

Correlations marked bold indicate strong correlations between similar dimensions of EQ-5D-5L and domains of SF-6D instruments (ρ ≥ 0.90) * *p* < 0.05, ** *p* < 0.01, *** *p* < 0.001.

## Data Availability

Data are available upon request by contacting the corresponding author.
